# Multiple bioinformatics analysis identifies IGFBP1 as associated with the prognosis of stomach adenocarcinoma

**DOI:** 10.1097/MD.0000000000033346

**Published:** 2023-03-31

**Authors:** Xiao-Ye Luo, Yan-Ping Zhang, Feng Zheng, Liang Zhou

**Affiliations:** a Surgical Department I, Hangzhou Lin’an TCM Hospital, Hangzhou, Zhejiang, China; b Department of Pathology, Hangzhou Lin’an TCM Hospital, Hangzhou, Zhejiang, China; c Surgical Department II, Hangzhou Lin’an TCM Hospital, Hangzhou, Zhejiang, China.

**Keywords:** prognosis recurrence, stomach adenocarcinoma, TCGA

## Abstract

This study aimed to screen the hub gene for predicting the prognosis of patients with stomach adenocarcinoma (STAD). The RNA-sequencing expression data and clinical data of STAD were collected from the cancer genome atlas. The R package “limma” was performed to ascertain the differentially expressed genes (DEGs) between the relapse group and non-relapse group, and the DEGs between the survival dead status group and survival alive status group were screened. The overlapping genes between 2 DEGs sets were identified by the Venn diagram. Many different bioinformatics analysis methods were performed to analyze the importance of hub genes. One gene signature, IGFBP1, was extracted. The KM plot indicated that STAD patients with low IGFBP1 mRNA expression have a shorter overall survival time. The top 100 co-expression genes of IGFBP1 were mainly enriched in complement and coagulation cascades, epithelial cell signaling in *Helicobacter pylori* infection, and Wnt signaling pathway. Immune infiltration analysis indicated IGFBP1 may inhibit immune cell infiltration in tumors by infiltration and immune escape, leading to tumor metastasis and progression. The bioinformatics analysis results indicate that IGFBP1 can be used as a tool to evaluate the mortality risk of patients with STAD.

## 1. Introduction

Based on Global Cancer Statistics 2020 which is an authoritative survey report to estimate incidence and mortality worldwide for 36 cancers in 185 countries by the GLOBOCAN, stomach adenocarcinoma (STAD) still is one of the most common malignant tumors worldwide. It is responsible for over 1 million new cases in 2020 and an estimated 769,000 deaths. The mortality rate is up to 1 in 13, ranking 4th for mortality globally after lung cancer, colorectum cancer, and liver cancer.^[1]^ Moreover, it was a notable finding that increased prevalence of stomach cancer in young people whose age is under 50 years.^[2,3]^ Some studies speculated that it was possible due to the abuse of antibiotics and acid suppressants.^[4,5]^

At present, radiotherapy, chemotherapy, tumor removal, immunotherapy, and surgery are considered effective for STAD in clinical, but the cure rate remains unsatisfactory because of its recurrence and metastasis.^[6]^ its prevalence of recurrence and metastasis exceeded 15% in groups with a combination of different clinical risk factors,^[7]^ to the extent that reduced the survival time of STAD patients, and increased the risk of tumor malignancy.^[8]^ Therefore, the determination of predictive biomarkers of STAD is extremely important. For instance, Zeng et al^[9]^ research showed that LCP1 was a prognostic biomarker correlated with immune infiltrate in gastric cancer. Hu et al^[10]^ study indicated that upregulation of PDLIM3 was significantly associated with poor prognosis in STAD. There are relatively few genetic markers based on recurrence-related. Hence, it is urgent to search for new recurrence and prognosis-related biomarkers for STAD.

In the present study, we aimed to screen for the hub genes that are prognostic and associated with recurrence based on the cancer genome atlas (TCGA)-STAD dataset by bioinformatics methods. As a result, insulin-like growth factor binding protein (IGFBP)-1 was screened. The relationship of its expression with clinical information was analyzed to assess its prognosis value. Moreover, its co-expression genes were collected from cBioPortal database, and the Kyoto encyclopedia of genes and genomes (KEGG)and gene ontology (GO) enrichment analyses were performed to research the potential function of IGFBP1. Analysis of immune cell infiltration levels was used to explore the relationship between the expression of IGFBP1 and the main 6 kinds of immune cells. All analyses indicated that it could be an independent prognostic factor for STAD.

## 2. Methods

### 2.1. Data source

**Figure 1.**
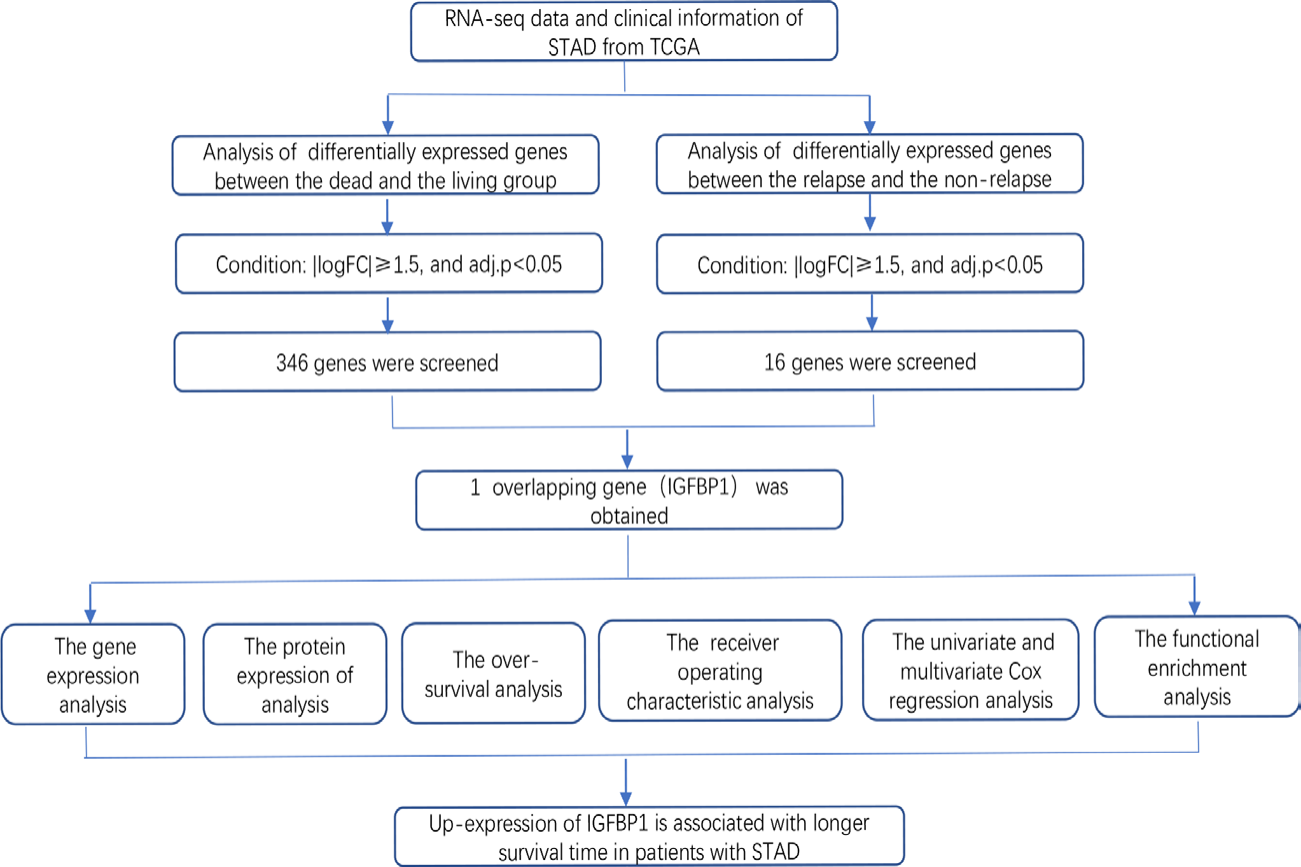
Flowchart of the data analysis.

The workflow for the present study is shown in Figure 1. The RNA-sequencing expression data and clinical data of STAD were collected from TCGA. Then, patients without overall survival and survival status were excluded, and a total of 392 samples including 357 STAD patients and 35 normal samples were analyzed. Moreover, the gene protein expression detected by immunohistochemistry (IHC) in tumor and normal tissue was explored from the human protein atlas and was verified by IHC experiment.

### 2.2. Differentially expressed genes identification

The STAD patients were collected from TCGA-STAD datasets. Then, those patients were grouped by relapse status and overall survival status. The R package “limma” was performed to normalize the RNA-seq expression data and ascertain the differentially expressed genes (DEGs) between the relapse group and non-relapse group with screening conditions including *P* < .05 and |log2FC|>1.5 in the TCGA-STAD datasets. The results were visualized by the ggplot2 R software package to draw a volcano plot, and the top 50 up-regulated and down-regulated genes were used as heat maps respectively. Analogously, the DEGs between the overall survival dead status group and overall survival alive status group were screened.

### 2.3. Functional enrichment analysis

The overlapping genes between 2 DEGs sets were identified by the Venn diagram. Then, their co-expression genes were collected from the TCGA database, and the top 100 co-expression genes were selected for further enrichment analysis. The “clusterProfiler” R package 15 was employed to perform KEGG analyses on the overlapping genes. *P* values were adjusted by the Bebjamini and Hochberg method. The (GO) function enrichment analysis also was performed, and cellular component, biological process (BP), and molecular function (MF) were annotated. These pathways were considered to be significantly enriched when the following criteria were met: nominal *P* value < .05, false discovery rate q value < 0.25, and absolute normalized enrichment score > 1.

### 2.4. IHC

IHC is a widely used diagnostic technique in tissue pathology. IHC use conventional 2-step staining. Tissues were embedded in paraffin, then sectioned and dewaxed to water. Antigen was repair by microwave heating. Section was washed with phosphate buffered saline and added primary antibody. After overnight incubation at 4°C, phosphate buffered saline was washed clean, and the second antibody was added and incubated at 37°C for 30 minutes. Then, section was colored by DAB. Hematoxylin re-staining, dehydration, transparency, sealing.

### 2.5. Statistical analyses

Kaplan–Meier method was performed to analyze the relationship between the expression of hub genes and the survival of patients with STAD, while the optimal cutoff point was adopted to divide patients into 2 groups, and the log-rank test was used to analyze the differences between survival curves. The receiver operating characteristic (ROC) curves were used to evaluate the prediction of hub gene expression on the survival probability by using the survival ROC package in R. Univariate and multivariable Cox regression analysis was used to assess the correlation between overall survival and hub gene expression in STAD patients with survival packages (version 3.6.4).

### 2.6. Analysis of immune cell infiltration levels

To research the relationship between the expression of the hub gene and 6 kinds of immune cells, we conducted the analysis of immune cell infiltration levels by the Tumor Immune Estimation Resource methods based on the TIMER2.0 database (https://cistrome.shinyapps.io/timer/) which was widely used to study immune cell infiltrates across various different tumors.^[11]^ The related immune cells included B cell, CD8 + T cell, CD4 + T cell, macrophage, neutrophil, and dendritic cell, and the relationship was corrected according to tumor purity. Moreover, the STAD patient was grouped into 2 groups by the expression level of the hub gene to research further correlations between the hub gene and the immune microenvironment score of 6 kinds of immune cells.

## 3. Results

### 3.1. Identification of overlapping genes between the 2 DEG lists

**Figure 2.**
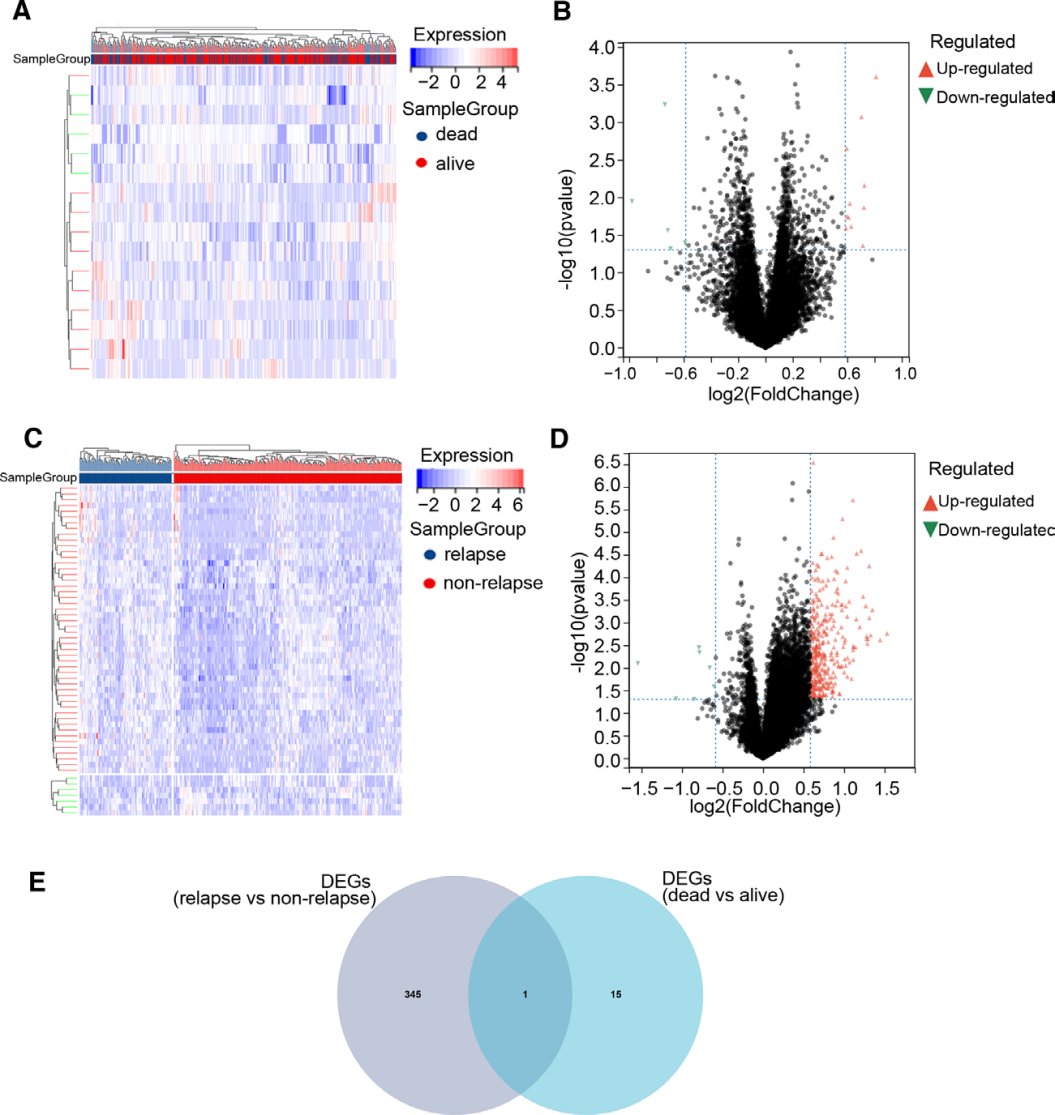
(A) Expression heatmap of DEGs between overall survival dead status group and overall survival alive status group. (B) Volcano plot of DEGs between overall survival dead status group and overall survival alive status group. (C) Expression heatmap of differentially expressed genes (DEGs) between the relapse group and non-relapse group. (D) Volcano plot of DEGs between the relapse group and non-relapse group. (E) The Venn plot of genes between 2 DEGs.

A total of 16 DEGs between the overall survival dead status group and overall survival alive status group were screened according to *P* < .05 and |log2FC|>1.5, including 11 up-regulated genes and 5 down-regulated genes (Fig. 2A and B). Similarly, 346 DEGs between the overall survival dead status group and overall survival alive status group were screened according to *P* < .05 and |log2FC|>1.5, including 339 up-regulated genes and 7 down-regulated genes (Fig. 2C and D). Then, 1 overlapping gene, IGFBP1, between the 2 DEGs was extracted by the Venn plot (Fig. 2E). This gene is a member of the IGFBP family and encodes a protein with an IGFBP N-terminal domain and a thyroglobulin type-I domain.

### 3.2. Differential expression of IGFBP1

**Figure 3.**
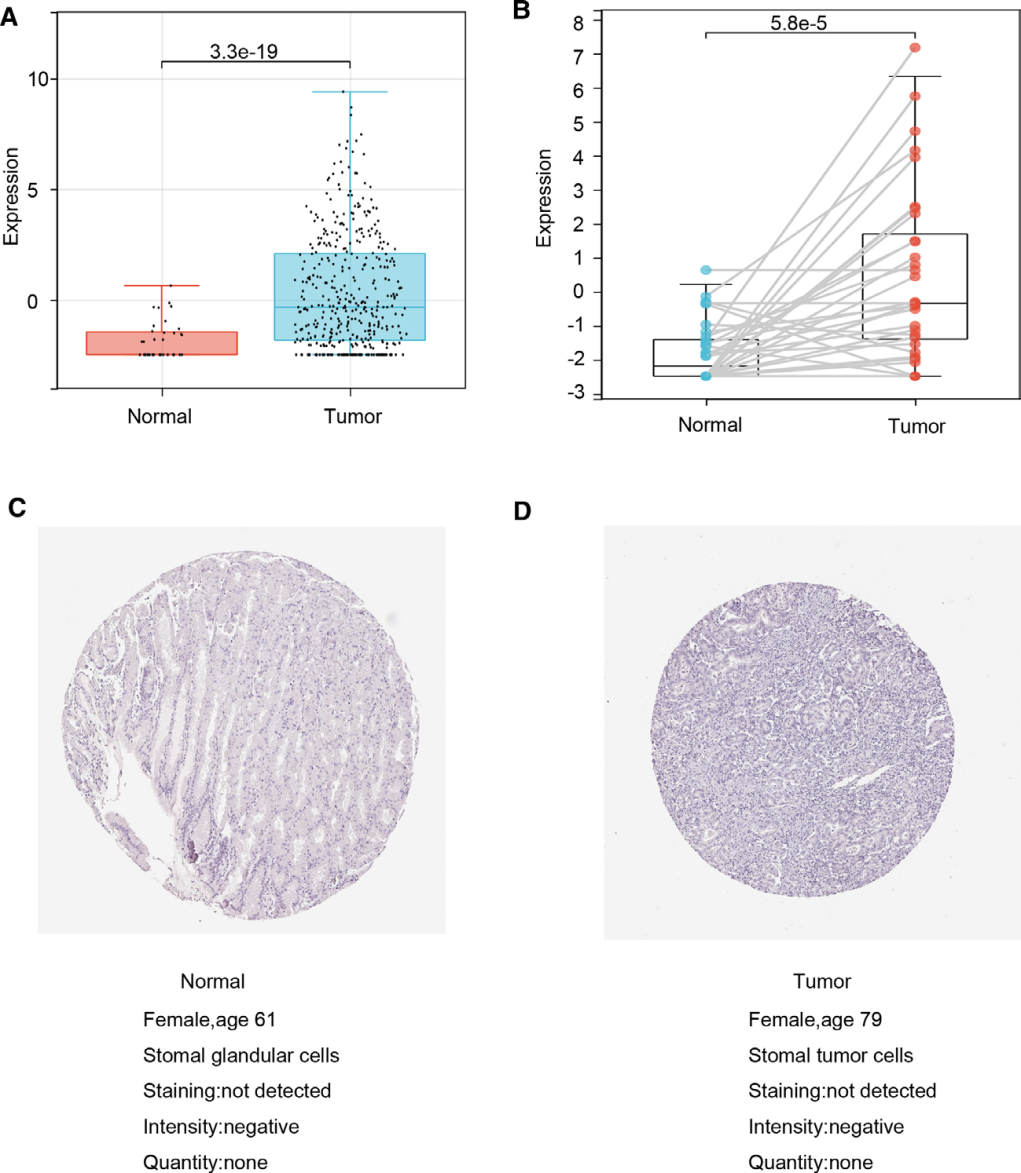
(A) Comparison of expression of IGFBP1 between STAD tissue and normal samples. (B) Comparison of expression of IGFBP1 between 35 paired samples. (C) Expression analysis of IGFBP1 protein in adjacent normal STAD tissues form HPA. (D) Expression analysis of IGFBP1 protein in STAD tissues from HPA. HPA = human protein atlas. IGFBP = insulin-like growth factor binding protein, STAD = stomach adenocarcinoma.

**Figure 4.**
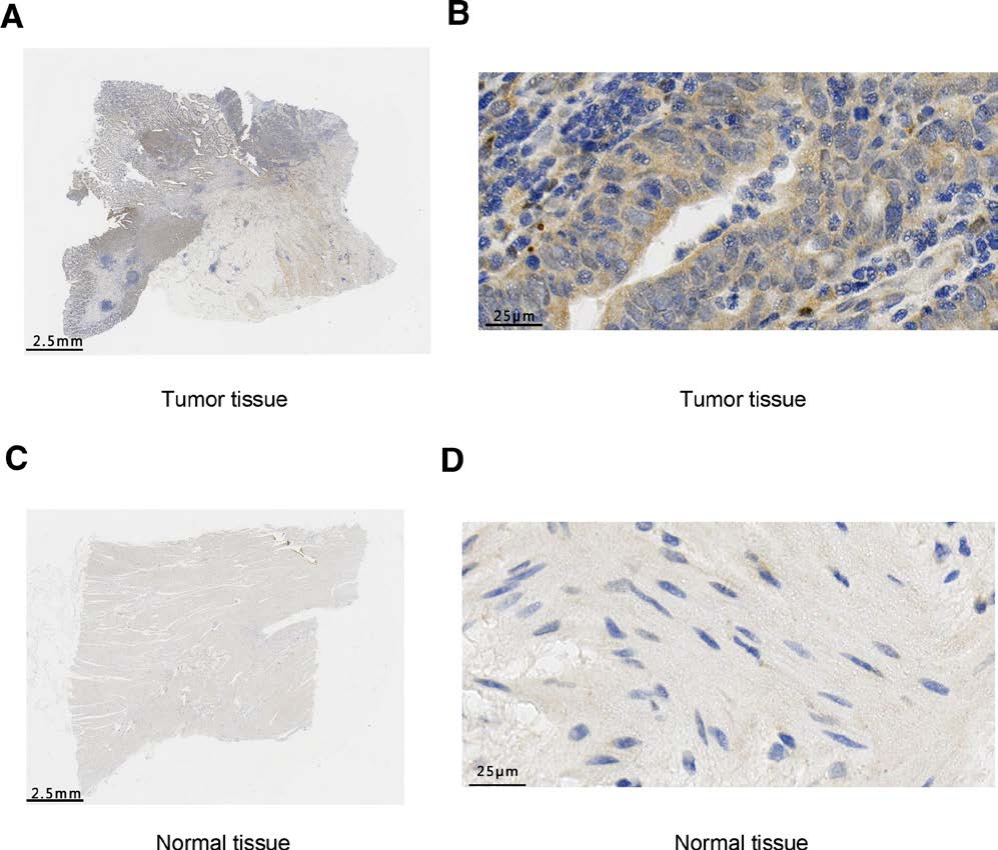
(A) The level of IGFBP1 protein in STAD tissue by immunohistochemistry, (B) magnification x80. (C) The level of IGFBP1 protein in normal tissue by immunohistochemistry, (D) magnification x80. IGFBP = insulin-like growth factor binding protein, STAD = stomach adenocarcinoma.

In order to reveal the clinical value of IGFBP1, we explored further its expression in STAD. The differential expression analysis was carried out. The result showed that its mRNA had a higher expression in STAD tissues than in normal tissues with significance both in TCGA-STAD datasets (Fig. 3A; *P* < .01) and paired samples (Fig. 3B; *P* < .01). Figure 3C and D showed that STAD protein expression by the immunohistochemical method was different between stomach cancer tissue and normal tissue according on human protein atlas database. Moreover, the level of IGFBP1 protein in STAD tissue was different with in normal tissue, which was verified by IHC experiment and the results are shown in Figure 4.

### 3.3. Survival analysis

**Figure 5.**
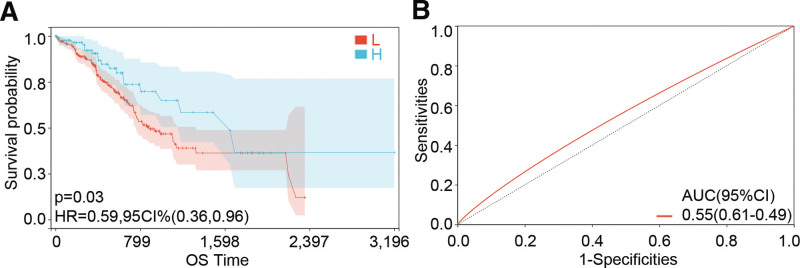
(A) Kaplan–Meier curves for IGFBP1expression in STAD. (B) ROC curves of IGFBP1 were evaluated according to OS. IGFBP = insulin-like growth factor binding protein, ROC = receiver operating characteristic, STAD = stomach adenocarcinoma.

The Kaplan–Meier analysis with the log-rank test was conducted to research the relationship between IGFBP1 expression and patients overall survival. The results indicated that STAD patients with low IGFBP1 mRNA expression have shown a shorter overall survival time (Fig. 5A). According to the ROC analysis result that area under the curve of receiver operating characteristic curve was 0.55, the expression of IGFBP1 had a prediction capacity on the survival probability of patients with STAD (Fig. 5B). To further explore the relationship between the expression of IGFBP1 and the overall survival (OS), the COX regression was carried out. The results indicated that the expression group of IGFBP1 was closely related to OS in both Univariable COX regression (HR: 0.62, 95% CI: 0.38–0.99, *P* = .04) and multivariate COX regression (HR: 0.59, 95% CI: 0.37–0.95, *P* = .03) while other clinical features did not relate to OS (Table 1).

**Table 1:** Univariate and multivariate Cox regression analysis of the IGFBP1 expression group and overall survival in STAD patients.

	Subject	B	*P* value	HR	Lower	Upper	Hazard Ratio(95% CI)
Univariable							
	Expression group	−0.49	.04	0.62	0.38	0.99	0.62 (0.38–0.99)
	Age	0.01	.14	1.01	0.1	1.02	1.01 (0.1–1.02)
	G	0.11	.48	1.12	0.82	1.52	1.12 (0.82–1.52)
	M	0.28	.13	1.32	0.93	1.88	1.32 (0.93–1.88)
	N	0.03	.65	1.04	0.89	1.2	1.04 (0.89–1.2)
	T	0.09	.39	1.1	0.89	1.35	1.1 (0.89–1.35)
	Stage	−0.17	.07	0.84	0.7	1.01	0.84 (0.7–1.01)
	Sex	0.21	.26	1.24	0.86	1.79	1.24 (0.86–1.79)
Multivariable							
	Expression group	−0.52	.03	0.59	0.37	0.95	0.59 (0.37–0.95)
	Age	0.01	.13	1.01	1	1.03	1.01 (1–1.03)
	Sex	0.2	.29	1.22	0.85	1.77	1.22 (0.85–1.77)
	Stage	−0.16	.1	0.85	0.7	1.03	0.85 (0.7–1.03)
	G	0.17	.29	1.18	0.87	1.61	1.18 (0.87–1.61)

IGFBP = insulin-like growth factor binding protein, STAD = stomach adenocarcinoma.

### 3.4. Functional enrichment analyses

**Figure 6.**
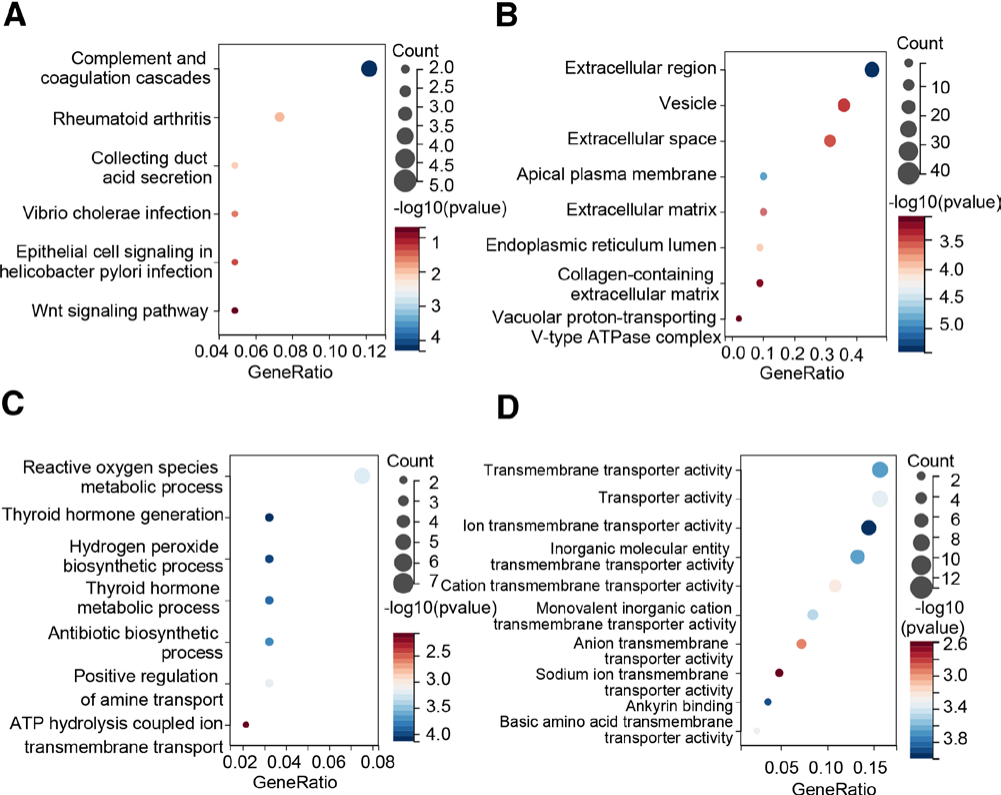
(A) KEGG pathway analysis of IGFBP1and top 100 co-expression genes in STAD. (B) Go enrichment analysis in STAD cellular component. (C) Go enrichment analysis IGFBP1of and top 100 co-expression genes of STAD in biological process and (D) molecular function. IGFBP = insulin-like growth factor binding protein, KEGG = Kyoto encyclopedia of genes and genomes, STAD = stomach adenocarcinoma.

Co-expression genes of IGFBP1 were downloaded from the cBioPortal database. KEGG enrichment analyses were conducted based on the top 100 co-expression genes to identify IGFBP1-related pathways and biological functions and significant pathways were shown in Figure 6A. The result showed that those genes were mainly enriched in complement and coagulation cascades, rheumatoid arthritis, collecting duct acid secretion, vibrio cholerae infection, epithelial cell signaling in *Helicobacter pylori* infection, and Wnt signaling pathway (Fig. 6A). The GO covers cellular components, BP, and MFs, and is widely adopted in functional enrichment analyses. In GO enrichment analysis, cell components were mainly engaged in the extracellular region, vesicle, extracellular space, apical plasma membrane, extracellular matrix, endoplasmic reticulum lumen, collagen-containing extracellular matrix, vacuolar proton-transporting V-type ATPase complex (Fig. 6B). The BP mainly involves reactive oxygen species metabolic process, thyroid hormone generation, hydrogen peroxide biosynthetic process, thyroid hormone metabolic process, antibiotic biosynthetic process, positive regulation of amine transport, ATP hydrolysis coupled ion transmembrane transport (Fig. 6C). Moreover, MF analysis showed that these genes were related to transmembrane transporter activity, transporter activity, ion transmembrane transporter activity, inorganic molecular entity transmembrane transporter activity, cation transmembrane transporter activity, monovalent inorganic cation transmembrane transporter activity, anion transmembrane transporter activity sodium ion transmembrane transporter activity, ankyrin binding basic amino acid transmembrane transporter activity (Fig. 6D).

### 3.5. Relationships between IGFBP1 and immune infiltration

**Figure 7.**
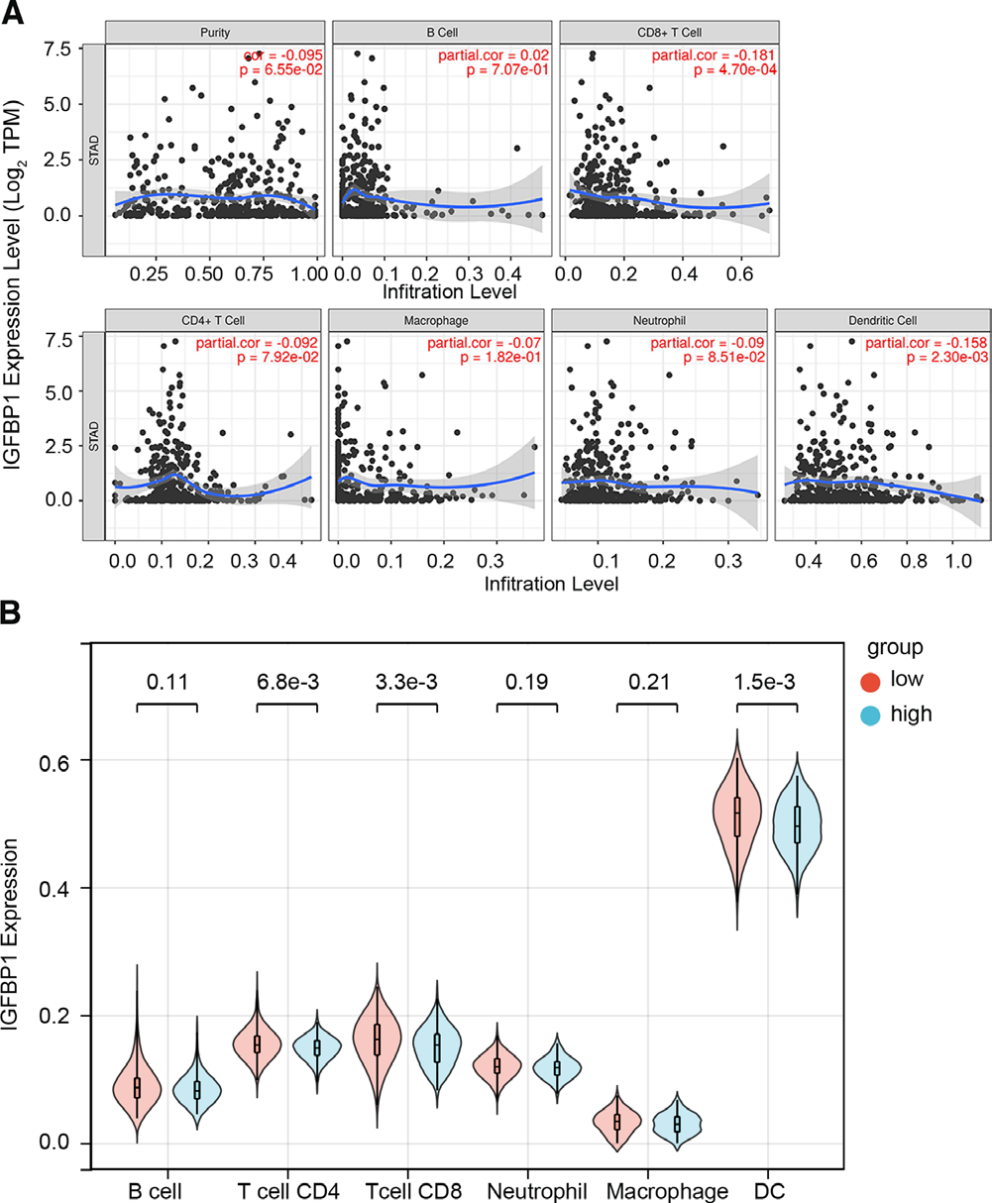
(A) Correlation scatter plot of IGFBP1 and 6 kinds of immune cells. (B) The difference in the infiltration of different immune cells in IGFBP1 high expression group and low expression group. IGFBP = insulin-like growth factor binding protein.

The results showed that IGFBP1 expression had a weak positive correlation with B cell infiltration, while it had a weak negative correlation with STAD tumor purity, CD8 + T cell, CD4 + T cell, macrophage, neutrophil, and dendritic cell. The Cor and *P* value was shown as follows B cell (Cor = −0.095, *P* = 6.55e-2), CD8 + T cell (Cor = 0.02, *P* = 7.07e-1), CD4 + T cell (Cor = −0.181, *P* = 4.70e-4), macrophage (Cor = −0.092, *P* = 7.92e-2), neutrophil (Cor = −0.09, *P* = 8.51e -2), and dendritic cell (Cor = −0.158, *P* = 2.30e-3) (Fig. 7A). However, the immune microenvironment score of the CD4 + T cell, CD8 + T cell, and DC was in the IGFBP1 low expression group significantly high than in the GFBP1 high expression group (Fig. 7B).

## 4. Discussion

In spite of various treatments, STAD remains a common malignant tumor of the digestive system with a high mortality rate.^[12]^ Moreover, the prognosis of patients with STAD is hard to predict individually using uniform clinical criteria due to its high heterogeneity. Therefore, it is necessary to find new biomarkers to perfect the prediction system for the prognosis of patients with STAD. The recurrence of STAD affects prognosis. However, fewer studies have focused on screening STAD prognosis biomarkers based on the recurrence-related gene. Therefore, in this work, DEGs between the overall survival dead status group and overall survival alive status group were screened based on TCGA-STAD datasets. Meanwhile, DEGs between the overall survival dead status group and overall survival alive status group were screened. Then, 1 prognosis and recurrence-related gene, IGFBP1, was extracted between the 2 DEGs.

IGFBP1 is a member of the IGFBP family and encodes a protein with an IGFBP N-terminal domain and a thyroglobulin type-I domain. It is rapidly metabolized in the circulation and insulin is the main regulator of its expression. The IGFBP1 family is able to regulate the activity of insulin-like growth factors (IGFs) by highly binding to them. IGFs is a multifunctional regulator of cell proliferation that promotes cell differentiation, proliferation, individual growth and development, and inhibits apoptosis.^[13]^ Moreover, IGF-1 was at a high expression level in STAD and promotes the proliferation and invasion of tumor cells through the JAK1/STAT3 signaling pathway.^[14]^ Recent in vivo studies have indicated that the IGFBP1 serves as a molecular switch restricting IGF signaling and diverts the limited energy resources from growth and development towards metabolic processes essential for survival under stress conditions.^[15,16]^ In the present study, we found that the IGFBP1 was a high expression in STAD tissue and its high expression was beneficial to the prognosis of patients with STAD. Therefore, we speculate that in STAD cells, IGFBP1 inhibits the IGF signaling pathway by binding to IGFs, thereby inhibiting cell growth. However, this result is in contrast to previously reported studies that high IGFBP1 expression was associated with hematogenous metastasis and poor survival due to different research patients. Specifically, all STAD patients with or without various treatments were included in this work, while only patients with gastric cancer who underwent gastrectomy were collected in the reported study.^[17]^

Luo et al^[18]^ have investigated that the expression and release of IGFBP1 were increased, with enhanced expression being associated with the migration ability of cancer cells in gastric cancer infected with *H pylori*, and suggesting that IGFBP1 may be a tumor-suppressor gene in the process of *H pylori*-induced STAD. Consistently, the KEGG enrichment analysis showed that the co-expression genes of IGFBP1 were related to epithelial cell signaling in the *H pylori* infection in the present study. *H pylori* infection is strongly associated with the development of STAD because *H pylori* infection can initiate inflammatory pathways in the gastric mucosa, and activate signaling pathways that relate the development and process of STAD. The previously reported studies have shown that *H pylori* improved ROS level and induced DNA damage in gastric epithelial cells. Moreover, the infection of *H pylori* activated P13K/AKT signaling pathways that were ROS mediated.^[19]^ In addition, *H pylori* virulence factor CagA can inhibit the proliferation of gastric cancer cells and promote the apoptosis of gastric cancer cells, and its mechanism may be related to the activation of the ERK signaling pathway by CagA.^[20]^ Patients with the same histological types of cancer may have different clinical outcomes due to different of immune cell infiltration.^[21]^ In this study, there was a negative association between the expression of IGFBP1 and CD8 + T cell, DC cell. In addition, the immune microenvironment score of the CD4 + T cell, CD8 + T cell, and DC was in the IGFBP1 low expression group significantly high than in the GFBP1 high expression group. These results suggest that IGFBP1 may inhibit immune cell infiltration in tumors by infiltration and immune escape, leading to tumor metastasis and progression.

## 5. Conclusion

In the present study, a gene biomarker of STAD was screened based on tumor recurrence-related and prognosis-related genes. The bioinformatics analysis results indicate that the gene signature can be used as a tool to evaluate the mortality risk of patients with STAD. However, this work remains some limitations due to the lack of experiments in vivo.

## Author contributions

**Conceptualization:** Xiao-ye Luo.

**Data curation:** Yan-ping Zhang.

**Formal analysis:** Feng Zheng, Liang Zhou.

**Writing – original draft:** Xiao-ye Luo, Yan-ping Zhang, Feng Zheng, Liang Zhou.

**Writing – review & editing:** Xiao-ye Luo, Yan-ping Zhang, Feng Zheng, Liang Zhou.
